# Advances in mouse genetics for the study of human disease

**DOI:** 10.1093/hmg/ddab153

**Published:** 2021-06-04

**Authors:** Steve D M Brown

**Affiliations:** MRC Harwell Institute, Harwell OX12 0RD, UK

## Abstract

The mouse is the pre-eminent model organism for studies of mammalian gene function and has provided an extraordinarily rich range of insights into basic genetic mechanisms and biological systems. Over several decades, the characterization of mouse mutants has illuminated the relationship between gene and phenotype, providing transformational insights into the genetic bases of disease. However, if we are to deliver the promise of genomic and precision medicine, we must develop a comprehensive catalogue of mammalian gene function that uncovers the dark genome and elucidates pleiotropy. Advances in large-scale mouse mutagenesis programmes allied to high-throughput mouse phenomics are now addressing this challenge and systematically revealing novel gene function and multi-morbidities. Alongside the development of these pan-genomic mutational resources, mouse genetics is employing a range of diversity resources to delineate gene–gene and gene–environment interactions and to explore genetic context. Critically, mouse genetics is a powerful tool for assessing the functional impact of human genetic variation and determining the causal relationship between variant and disease. Together these approaches provide unique opportunities to dissect *in vivo* mechanisms and systems to understand pathophysiology and disease. Moreover, the provision and utility of mouse models of disease has flourished and engages cumulatively at numerous points across the translational spectrum from basic mechanistic studies to pre-clinical studies, target discovery and therapeutic development.

## Introduction

For well over a century, comparative biology has been a considerable force in our understanding of mammalian physiological systems and by translation our understanding of pathological and disease mechanisms. The similarities and dissimilarities of physiology in diverse mammalian species have thrown much light in particular on human physiology where invasive studies are precluded. Comparative biology has been newly enriched by the genomics revolution, which provides an evolutionary perspective on the emergence of diverse biological mechanisms, and has enabled the development of genetic tools to enhance comparative biology and most importantly provide a genetic perspective to the understanding of physiological and disease systems.

**Figure 1:**
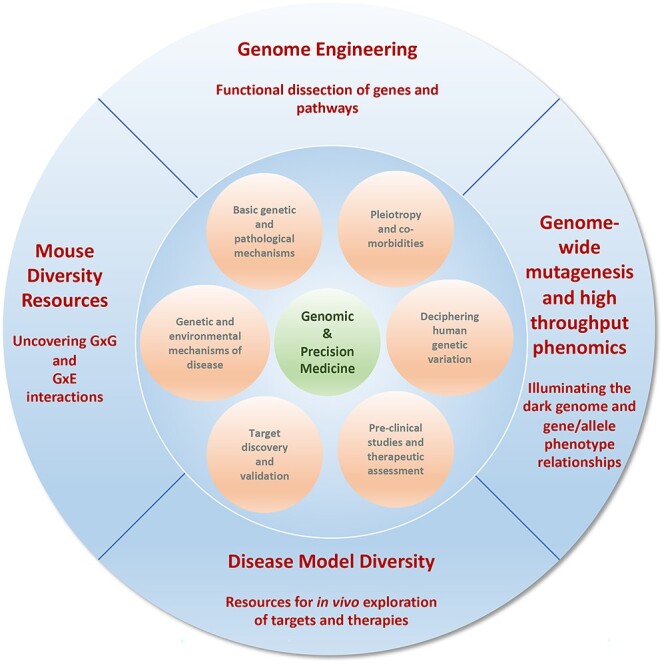
Charting the key routes by which mouse genetics and genomics approaches (outer circle) contribute to the landscape of mechanistic, systems, pathobiology, disease genetics, pre-clinical and therapeutic studies (inner circle) that will be critical to address the challenges of genomic and precision medicine.

A number of key model organism systems have emerged to exploit this genomics revolution, but within mammals, the mouse has been pre-eminent. Over the last 30 years, mouse genetics, biochemical and physiological studies have transformed comparative biology in mammals, and importantly, provided a fundamental basis for insights into human physiology and disease mechanisms. A critical goal for genomic medicine, therapeutic discovery and healthcare is to deliver a profound understanding of the causal relationship of genetic variation and disease, including gene–gene and gene–environment interactions. This article reviews the major developments in mouse genetics and genomics that have contributed to this goal and the advancement of our understanding of human disease and the role of genetic variation, and sets a course for future contributions of the mouse from genomic medicine to healthcare.

## Gene and Phenotype

A central theme of mouse genetics over the last few decades has been to garner tools and resources and harness these to the development of approaches to investigate the relationship between gene and phenotype (Fig. 1). Fundamentally, the elucidation of gene–phenotype mechanisms in the mouse underpins our understanding of the genetic bases for disease and enables the dissection of the interplay of pathological and physiological mechanisms.

In the 80s and 90s, the available resources in the mouse to explore how genetic variants impact upon phenotype were relatively limited. Since the early part of the 20th century, numerous inbred strains of mice had been developed providing a diversity resource, which was later to be exploited for the study of multi-factorial disease (see Uncovering Genetic Systems in Mouse and Human - The Utility of Mouse Diversity Resources). In addition, over the course of the last century, a large collection of spontaneous mouse mutants was identified and described. In the 90s, with the application of molecular genetics approaches to positional cloning in mouse and humans, the identification of the underlying genes for a number of these mutants demonstrated the power of mouse genetics to unleash novel insights into the gene–phenotype relationship. Examples include the discovery of leptin, which identified a new hormone and opened up fresh vistas in metabolism and appetite control (1); the first insights into the molecular machinery of auditory transduction, throwing light on the genetics of human deafness (2,3); and the identification and characterization of the *mdx* mutant, which played a foundational role in the study of the mechanisms of muscular dystrophies (4).

A key realization was the power of classical forward genetics (or phenotype-driven genetics) to uncover novelty in the gene–phenotype relationship that cannot be discerned by the examination of gene sequence alone or by *in vitro* methods. Importantly, this also applies to reverse genetics (or gene-driven genetics), when applied at scale and in an unbiased fashion, to ask genome-wide questions about the relationship of genes and genetic pathways to phenotype (and by consequence to disease). In both scenarios, the aim is to set aside a priori assumptions about the role of genes in disease in order to uncover novelty in the gene–phenotype space (5).

## Genome Engineering in the Mouse—Tools for the Dissection of Genes and Pathways

Following the identification of embryonic stem (ES) cells in the mouse and the demonstration of their manipulability for the generation of mice carrying null, knock-out mutations, there has been a flowering of tools generated to dissect the function of specific genes (6). The ability to generate alleles carrying genetic elements knocked into specific genes or chromosome locations has had a profound impact on our understanding of genetic and disease mechanisms. For example, Cre-recombinase systems (or the alternative Flp) employing Cre-driver resources (7) for the conditional ablation of gene function are utilized to explore gene function at specific times during development and in specific tissues and cell types (8). In addition, the tetracycline/doxycycline-regulated Tet-On and Tet-Off binary systems with a transcriptional transactivator as a driver allow quantitative and temporal regulation of a target transgene (9).

Multiple mouse lines carrying reporters introduced into specific genes have been generated to follow gene expression throughout the life course. The Brainbow technology provides a powerful application of fluorescent transgenes for diverse investigations such as axon tracing and cell lineage analysis (10). A sophisticated extension of these approaches is the development of mouse lines carrying various optogenetic or chemogenetic actuators, which along with the binary utilization of Cre driver and actuator lines has transformed studies of neuronal function in specific cell types and regions of the brain (11). In addition, combinations of Cre and Flp drivers, for example, enable intersectional approaches to further enhance the specificity of analysis with regard to cell type (12).

Importantly, there is an increasing focus on ES cells and Clustered Regularly Interspersed Short Palindromic Repeats - CRISPR associated protein 9 (CRISPR-Cas9) technologies for the humanization of the mouse genome to explore the comparative function of individual genes, their pathways and associated genetic variants (13). This includes humanization of specific amino acids, where individual codons are changed (14); the humanization of individual domains and exons in a target gene (15); the complete humanization of a mouse gene, often retaining the regulatory context of the mouse (16); and humanization of extensive genomic regions, such as the immunoglobulin loci (17). Although it is sometimes expedient and easier to utilize CRISPR-Cas9 in ES cells, for many aspects of humanization from targeted coding mutations to the humanization of gene domains and even whole genes, CRISPR-Cas9 approaches in zygotes are available. They are the preferred choice for introducing targeted coding mutations. CRISPR-Cas9, or alternatively ES cells using the Cre-lox system, have been used to develop substantial megabase duplications and deletions, extending over significant chromosome regions, allowing for the modelling of human copy number variants and aneuploidies in human (18,19). In addition, the development of transchromosomic mice was founded on the manipulability of ES cells and the introduction of human chromosome material into mice (20).

The toolbox is seemingly boundless, but while these approaches enable an extraordinarily fine and detailed genetic analysis of the function of individual genes or chromosome disorders, they do not of themselves provide scalable routes to the pan-genome cataloguing of gene function—in particular, a baseline catalogue of mammalian gene function developed from a genome-wide null mutation resource of the mouse. A first step towards such a goal was taken by the International Knockout Mouse Consortium (IKMC) with the development of a genome-wide knock-out first conditional ready library of mutant ES cell lines (21). In addition, the more recent development of CRISPR-Cas9 technologies for the rapid and scalable generation of targeted and complex mouse mutations (22,23) brings a new dimension to these challenges and the development of disease models (24), which we discuss below.

## Genome-Wide Mutagenesis Approaches—Gene Function Analysis at Scale

The earliest programmes that set out to tackle genome-wide analysis of gene function employed phenotype-driven approaches, by and large N-ethyl-N-nitrosourea (ENU) mutagenesis (25,26), but in some cases, transposon mutagenesis (27). ENU, an efficient chemical mutagen, introduces random point mutations across the genome. Phenotypic assessment of pedigrees from mutagenized mice allows the recovery of both dominant and recessive mutations (usually missense coding variants) for which the underlying genes can be identified through mapping and sequencing. The unbiased and hypothesis-free nature of this mutagenesis approach proved successful in identifying a large number of novel genes underlying the detected phenotypes often uncovering novel mechanistic insight across a diversity of biological systems. For example, the role of primary cilia in hedgehog signalling in mammals was largely unravelled through ENU screens in mice (28).

ENU mutagenesis programmes highlighted for the first time the value and efficiencies of high-throughput phenotyping pipelines whereby individual mutant mice are assessed across a range of phenotypic domains that inform on models for diverse diseases. Many ENU screens were adapted to employ phenotyping pipelines that search for a range of phenotypes covering a variety of disease states, uncovering for each disease area a new range of mutant phenotypes and their underlying genes. Such a pipeline, for example, was used in a recessive ENU ageing screen whereby cohorts of ENU mutagenized mice were recurrently screened through the phenotyping pipeline at various timepoints as the mice aged (29). A significant number of mutants and their novel underlying genes were identified that would not have been uncovered by screens at earlier timepoints.

## The Dark Genome and Pleiotropy—The Development of High-Throughput Mouse Phenomics

Notwithstanding the impact of these extraordinary developments in mouse genetics and genomics tools and their early application to genome-wide analysis of gene function, the function of the majority of genes in both the mouse and human genomes remains unknown or poorly described. Progress in genomic and precision medicine is threatened by the ‘dark genome’ and the absence of a comprehensive catalogue of mammalian gene function (30,31). The dark genome not only refers to the minimal knowledge on biological function available for many genes, but also to the limited resources and tools (such as antibodies) for their analysis. Despite two decades of discovery science following the publication of the human and mouse genome sequences, the dark genome endures. The persistence of the dark genome is partly reflected in recent analyses, which find that genes studied in the past tend to be those studied in the future (32). There is an inherent tendency for work and funding to be disproportionately focused on genes for which there is prior knowledge. However, set against this, Stoeger *et al*. (32) find that studies of poorly researched human genes are primed and aided by large-scale model organism studies.

Uncovering pleiotropy is also compromised by the dark genome (31). Pleiotropy, the multiple functions of a gene, manifests as multi-morbidities in disease. Pleiotropy is ubiquitous in plants and animals and is revealed through Genome-wide Association Study (GWAS) and Phenome-wide Association Study (PheWAS) analyses in humans, and also in the phenotyping analysis of mouse models. Nevertheless, it is not possible to ascertain complete phenotype information pertaining to each gene locus from human studies. In addition, for many extant mouse models, where the phenotyping often reflects the interests and expertise of the investigator, pleiotropy, to its fullest extent, is rarely revealed. Our limited knowledge of pleiotropy is another aspect of the dark genome. Yet, the identification of co-morbidities for genetic disease will be vital in terms of mechanistic studies, diagnosis and therapeutic strategies.

Tackling the dark genome and uncovering pleiotropy requires the development of high-throughput broad-based phenomics in the mouse that is applicable at scale to the entire genome. Moreover, the pipelines should be standardized and assessed to ensure robustness and reproducibility across time and place. The EUMORPHIA programme developed and assessed a set of standard operating procedures, which are available as the EMPReSS database (33). By incorporating appropriate metadata (such as apparatus, environment, feed, experimenter, time of test), the phenomics pipeline also takes account of experimental confounders and gene–environment interactions. The EMPReSS phenomics pipeline covers a very wide range of disease states including neurological and behaviour, metabolism, cardiovascular, pulmonary, sensory, musculoskeletal, immune and reproduction. A constant drive to deliver improvements in the breadth and efficiency of mouse phenotyping pipelines is imperative, including humanizing the test environment, particularly for behavioural assessment and the application of automated home cage analysis (34–36). Critically, all phenotyping pipelines should take account of sex, including appropriately powered cohorts of female and male mice to ensure that sex-specific effects can be determined and sexual dimorphism uncovered (see Genome-Wide Analysis of Gene Function and Pleiotropy Using High-Throughput Phenomics). In addition, it is recognized that age may be a key determinant of phenotype, and that incorporating cohorts of ageing mice into phenotyping pipelines has the potential to uncover novel phenotypes not detected at earlier timepoints (see Genome-Wide Mutagenesis Approaches - Gene Function Analysis at Scale) (29).

## Genome-Wide Analysis of Gene Function and Pleiotropy Using High-Throughput Phenomics

The availability of a genome-wide ES cell library of knock-out first, conditional ready mutations from the IKMC (7,21) allied to robust and reproducible high-throughput phenotyping pipelines provided the foundation for systematic genome-wide analysis of gene function. Two pilot programmes were established to first assess the feasibility of this approach. The Mouse Genetics Project at the Sanger Institute undertook the generation of hundreds of mutant lines analysed through a range of phenotyping screens (37). The European Mouse Disease Clinic programme embarked on a multi-centre programme also generating hundreds of mutant lines analysed through the EMPReSS phenomics pipeline (38). Both programmes found a remarkable depth of pleiotropy across the mutants analysed. Importantly, these pilots demonstrated the potential for scalability of these approaches for a genome-wide analysis. It has been argued that null mutations, which do not reflect the bulk of genetic variation in the human genome, are a poor tool for dissecting the genetic of disease susceptibility. However, a major analysis of the human population uncovered thousands of associations between Mendelian and complex disease that form a phenotypic code linking Mendelian loci and complex disorders, underlining the relationship of loci carrying loss-of-function mutations to complex disease and the utility of their analysis (39).

In 2011, the International Mouse Phenotyping Consortium (IMPC) was formed and adopted these approaches with the aim of generating a complete catalogue of mammalian gene function, importantly placing all resources and data in the public domain to ensure accessibility and utility. The IMPC encompasses 21 research centres worldwide (mousephenotype.org). The IMPC initially generated a null mutant for each gene from the IKMC library (40), a portion of null mutants deriving from alleles created by Regeneron (41). However, the IMPC now utilizes faster, cheaper CRISPR-Cas9 to produce null alleles by deletion of an early critical exon in each gene. In all cases, mutants are generated on a C57BL/6N isogenic background. Robust protocols are in place to confirm the genotype of each mutant, creating a gold-standard resource. Cohorts of male and female homozygous mutant mice are generated (7 + 7) and enter the early adult IMPReSS (successor of EMPReSS) pipeline followed by a variety of terminal tests. Homozygous embryonic lethals undergo phenotyping through an embryonic phenotyping pipeline that assesses lethality timepoints and morphological analysis utilizing various high-resolution imaging modalities. For homozygous lethals, the heterozygotes enter the adult phenotyping pipeline. Utilizing the lacZ reporter that is incorporated within the IKMC allele, IMPC has been able to capture expression data in both embryos and adults for many of the genes analysed. Worldwide data from IMPC centres is downloaded nightly to the Data Coordination Centre for IMPC, where robust quality control (QC) analysis is undertaken prior to the application of statistical analysis for the identification of significant phenotypes, before deposition in the Core Data Archive at the European Bioinformatics Institute (EBI). All IMPC data and biological resources are open access.

At the beginning of 2021, the IMPC had generated null mutations for 9719 mouse<->human orthologous genes, representing over half the orthologous genome. Of these, 7455 have been phenotyped through the IMPC comprehensive phenotyping pipeline. In total, across the worldwide community, null mutations have been generated for 13 657 genes, of which 5646 are exclusive to IMPC. There remain 3999 orthologs without mouse null mutations, which are a target for future work of the IMPC.

The current multi-dimensional dataset from IMPC encompasses nearly 100 million datapoints and 520K images. The analysis of these data as they emerged has demonstrated the power of this approach to uncover novel gene function and provide a large new fund of models for human disease. An early analysis of the first 3328 genes in 2017 demonstrated that for over half of these genes, this was the first mutant created. Moreover, across the entire dataset, 90% of gene–phenotype annotations had hitherto not been reported (42). A total of 889 known human disease genes were found to have an IMPC mutant with at least one phenotype, of which 360 lines (40.5%) had a phenotypic overlap with the human disease. For the majority of these lines, 279 (78%), this is the first reported mutant of the human disease. A further analysis of the data in 2019 encompassing 4736 genes (43) found a similar picture with now 1484 mutants with significant phenotype annotations and a human disease ortholog. Of these, 615 (41.4%) mimic the human phenotype.

The broad spectrum of phenotypes assessed in the IMPC pipeline has enabled studies of potential genes involved in specific disease domains that have remained hidden or previously not investigated. Pan-genomic analyses of the IMPC dataset for a number of disease areas have now been reported, including metabolism (44), deafness (45), eye development (46), circadian rhythms (47) and bone (48). Each of these uncovers an extensive and unexplored landscape of novel genes and mechanisms involved with each disease domain, and further underscores the significant insights into the dark genome. Moreover, the analysis of the large number of embryonic lethal lines uncovered has provided an important window into the nature of essential (lethal) genes in both human and mouse and their relationship to disease (49). Many of the mouse lethal genes are also found to be essential in human cell lines and are designated ‘cellular lethal’, in contrast to the remaining lethal genes, which are not associated with cellular lethality and are classified as ‘developmental lethal’ genes. This class of ‘developmental lethal’ genes is highly enriched for human disease genes (50). Finally, the analysis of the IMPC dataset has demonstrated the pervasive and wide-ranging sexual dimorphism of phenotypic traits in both wild-type and mutant mice (51) with profound implications for precision medicine.

It is important to reflect that phenotypes have only been assessed on one genetic background, and clearly there are further genetic effects that remain to be revealed in additional genetic contexts (see Uncovering Genetics Systems in Mouse and Human - The Utility of Mouse Diversity Resources). Notwithstanding this, for many genes, the resource provides the only available insights into gene function. In summary, global, genome-wide gene function programmes are illuminating the dark genome, uncovering deep knowledge on basal gene function, pleiotropy and co-morbidities, and providing a large and novel fund of mouse models of disease.

## Deciphering Human Genetic Variation—The Role of Mouse Genetics

A critical challenge for genomic medicine is to determine the causal relationship between human genetic variants and disease. Completing a catalogue of mammalian gene function provides a fundamental platform to assist with deciphering which genes are associated with particular diseases. Nevertheless, it is important to refine approaches to deliver a more exquisite understanding of the relationship between specific human variants and disease. Across rare diseases and Mendelian disorders, we are faced with a large number of variants of uncertain significance in both known and unknown disease genes (52). The mouse provides a critical path for ascribing a specific gene or variant to a disease (53,54). Mouse genetics centres worldwide have already established CRISPR pipelines for the generation of mice carrying human disease-associated coding variants. These include the various Rare Disease Models and Mechanisms initiatives across the globe (55) (https://j-rdmm.org; http://solve-rd.eu/rdmmeurope/). The phenotyping of these mutants will test the validity of the variant in question. Equally, comprehensive phenotyping of these mutants can reveal pleiotropy, extending our understanding of co-morbidities and improving diagnosis and potentially treatment. Moreover, these pipelines provide important models for pre-clinical testing and therapeutic development.

Such pipelines might be extended to the analysis of genetic variants associated with non-coding sequences, which are increasingly recognized as an important contribution to human disease (56). The challenge of scaling the analysis to tackle the potentially huge array of variants in non-coding elements (CNEs) is formidable, but an initial focus on conserved CNEs would be a compelling start point.

Already mouse pan-genome functional data, principally from null mutations, is having a considerable impact on deciphering the significance of human variants. Data integration and ontology developments across mouse and human are critical, along with the appropriate software tools (57,58). Automated variant prioritization tools that compare clinical phenotypes to those observed in the mouse, such as Exomiser, are proving invaluable. Inclusion of mouse and fish model phenotype data in Exomiser increases the validation rate of variants by 10–20% in the National Institutes of Health Undiagnosed Disease Programme (59).

**Figure 2:**
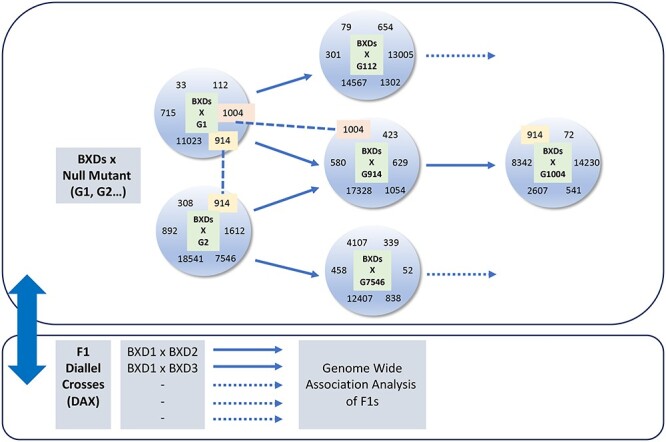
Integration of mouse mutant and mouse diversity resources will illuminate the complexities of disease genetic networks. In this example, pan-genomic null mutant resources, such as IMPC [G1, G2…] (40) are crossed to a set of BXD inbred lines (a BXD/G cross) to identify a number of modifier loci (annotated within the circles). The identified modifier loci become targets for further rounds of BXD/G crosses where the null mutant of a modifier locus is crossed to the BXD set. Common modifier loci identified in more than one BXD/G cross would be priorities for further BDX/G analysis, as exemplified by the modifier loci 914 and 1004 highlighted in the figure. In parallel, the large-scale generation and phenotyping of F1 diallel crosses (DAX) from BXD lines enables a powerful analysis of epistasis and dominance providing the basis for complex genetic models of the genetic pathways uncovered (62).

## Uncovering Genetic Systems in Mouse and Human—The Utility of Mouse Diversity Resources

The genetic context (G × G interactions) and environmental context (G × E interactions, including ageing) will impact greatly on gene–phenotype and gene–disease relationships. Although genome-wide mutation resources provide an important baseline for fundamental knowledge on gene function and disease, they are unable to capture straightforwardly the phenotypic consequences of genetic background, though environmental impacts can be factored into phenotype platforms. In order to unravel the complexities of genetic context, mouse genetics provides a variety of resources built from the available diverse inbred mouse lines, including a number of wild-derived inbred lines (60,61).

Beyond the mouse inbred lines themselves, there are two classes of tools. First, there are the recombinant inbred (RI) lines, each line isogenic, and which include the BXD recombinant inbred panel (62) and the Collaborative Cross (CC) lines (63). The BXD lines encompass 6 million common sequence variants (more than many human populations) and provide high resolution for the mapping of quantitative trait loci (QTLs). They have been phenotyped over many years for a range of phenotypes, including >100 omics datasets and >7500 classic phenotypes. The number of BXD strains has recently been expanded, providing more power and resolution (62). Importantly, crossing BXD lines together to generate a diallel cross (DAX) of BXD F1 lines provides further enhancements to map epistasis and evaluate complex genetic models as well as study dominance. Moreover, BXDs can be crossed to single gene models to map modifiers of disease traits. In an elegant demonstration, Neuner *et al*. (64) generated a panel of isogenic F1s generated by crosses between the 5XFAD Alzheimer’s disease (AD) model and 27 BXD lines. Not surprisingly, cognitive phenotypes varied widely across the F1 strains, reflecting the variability seen in the human population and likely mirroring genetic variants that segregate in the BXD reference population. They computed a genetic risk score for each strain based on 21 human genes that increase human AD risk and showed that allele dosage at these loci was significantly associated with cognitive outcomes across the 27 lines. Thus, the modifying effects of genetic background across the BXDs recapitulate the effects of genetic context in the human population. Extending the number of lines used will allow investigators to identify further loci, refine mapping and identify underlying loci that modify AD, and in particular loci that contribute to resilience. Importantly, the incorporation of diversity into model systems not only elaborates the complexity of the genetic pathways involved and their interaction but also provides an improved platform for pre-clinical testing of personalized therapeutic interventions.

The CC lines have also been studied for a number of disease phenotypes and also provide high-resolution mapping capabilities, which has been utilized to examine the genetic bases for a number of traits (65,66). In addition, the Hybrid Mouse Diversity Panel, which combines inbred strains and RI strains for the mapping of QTLs, has also been employed to localize diverse traits (67,68).

A second class of mouse tools that are used for QTL analyses involves the generation of outbred populations by pseudo-random breeding schemes. These include Heterogeneous Stocks (HS) (69) and Diversity Outbred (DO) Populations (70). HS mice are created by the intercrossing of inbred or recombinant inbred lines followed by mating schemes that minimize inbreeding. A form of HS, the DO population, is generated by random mating of partially inbred CC lines. Both types of outbred populations have been utilized to map disease traits (71,72). Finally, commercially available outbred populations have been used for mapping of specific disease traits (73) and, coupled to high-throughput phenotyping pipelines, have also been employed at scale for discovery and mapping of a wide range of disease loci (74).

Integration of diversity resources in the mouse with large-scale efforts to develop and characterize null mutant (and other monogenic alleles) resources, such as IMPC, will be critical to fully exploiting all of these advances. Single gene mutant resources, comprehensively phenotyped, are fundamental to transitioning from trait loci to causal genes, providing important validation and biological insight. Indeed, they are an important substrate, as exemplified above, for combining resources such as BXDs with disease models to investigate modifier effects on a systematic basis, gene by gene. Under such a scheme, each mutant would be crossed to a standard set of BXDs (a BXD/G cross) and modifier loci identified (Fig. 2). These modifier loci themselves become targets for further rounds of analysis where a null mutant at the modifier gene becomes the target for a further round of BXD/G analysis. As this iterative process proceeds, common modifier loci between BXD/G crosses would begin to elaborate genetic networks—and indeed such common modifiers would themselves be a priority target for further BXD/G analysis. Coupled with the F1s generated from diallel crosses of BXD lines (62), a plethora of networks related to pleiotropy and disease mechanisms would be revealed. The scale of this enterprise would require substantial investment and an international effort, but would be a decisive step in unravelling the genetic networks of disease and underpinning genomic and precision medicine.

## Translating Findings in Mouse Genetics to Drug Target Discovery, Therapeutic Development and Precision Medicine

In 2003, in a seminal paper, Zambrowicz and Sands (75) undertook a retrospective review evaluating the phenotypes of mouse knock-outs of targets for the 100 best-selling drugs. They uncovered a good correlation of phenotypic effects with known drug efficacy underlining the utility of the mouse for target discovery and disease modelling (75). The potential of mouse genetics approaches and resources for therapeutic development and healthcare remains no less compelling today. Indeed, a recent analysis of gene–disease association data from the Open Targets platform demonstrated that one of the datatypes with the best predictive power for novel therapeutic targets was animal model disease–phenotype data (76). Nevertheless, there has been frustration at the apparent failures of pre-clinical studies to translate to humans (77). The biomedical sciences community has seized the opportunity to tackle the underlying challenge of validity and reproducibility, which are a key to the utility of mouse models, including characterization and selection of models, controlling variation and its role in phenotypic outcomes, as well as appropriate statistical tools. From this perspective, it is important to recognize that the critical role of any model in the translational engine may engage at various points across the spectrum of activities that lead to improved healthcare—including basic mechanistic and pathophysiological studies, pre-clinical studies and target discovery, and therapeutic development (Fig. 1). In addition, in this context, it is useful to disregard the strict notion of ‘predictive validity’ often employed with relevance to drug responses in model organisms (78). As discussed below, mouse mutants play diverse and critical roles in the journey from pathophysiology to target to therapy. A number of examples serve to illustrate this point.

The immediate concerns relating to coronavirus disease 2019 (COVID-19) provide a compelling illustration of the power of mouse models to inform on disease pathology and treatment. Zheng *et al*. (79) have utilized a humanized transgenic mouse for the Angiotensin Converting Enzyme 2 (ACE2) receptor (K18-hACE2), originally developed for exploring severe acute respiratory syndrome infection, and characterized body-wide responses to severe acute respiratory syndrome coronavirus 2. Infected mice develop dose-dependent lung disease along with anosmia, and in some cases brain disease. Pre-treatment with convalescent plasma from human patients prevented severe disease but not anosmia. K18-hACE2 is thus a powerful model to study disease mechanisms and pathology, as well as for the assessment of therapeutic interventions. Notwithstanding these achievements, the development of additional COVID-19 susceptible mouse models that develop milder disease outcomes would be advantageous.

Recent analysis of a novel *Kctd13* null mutant, which lies within the 16p11.2 region associated with autism spectrum disorder and disability uncovered a range of cognitive phenotypes similar to mouse deletion mutants covering the homologous region (80). Together these data indicated that the Ras homolog family member A (RHOA) pathway is involved, and chronic treatment with fasudil (HA1077), an inhibitor of the Rho-associated protein kinase, restored object recognition memory along with learning and memory improvements in adult heterozygous mutant mice for *Kctd13* and the 16p11.2 deletion. These pre-clinical studies identify a new target gene within the 16p11.2 region for autism spectrum disorder along with a potential new therapeutic avenue.

Equally, mouse mutant resources can be utilized to identify new therapeutic strategies for known disease genes, exemplified by the analysis of Wolfram syndrome characterized by childhood-onset insulin-dependent diabetes mellitus and progressive optic atrophy with an underlying endoplasmic reticulum (ER) disorder. Two causative genes, Wolfram syndrome 1 and Wolfram syndrome 2, have been described, but identification of druggable targets has proved problematic. Recent work, employing cellular studies as well as both *Cisd1* and *Cisd2* mouse mutants, has established the molecular mechanism as calpain hyperactivation and a new target pathway (81). A small-scale screen for molecules targeting ER calcium homeostasis identified dantrolene, which prevented cell death in patient induced pluripotent stem cells (iPSC) derived neural progenitor cells and also suppressed calpain activation in brain lysates from brain-specific *Cisd1* knock-out mice.

Finally, there are numerous examples of the use of mouse models to explore and assess gene therapy approaches. These include the treatment of recessive genetic disorders where gene replacement strategies can be employed to restore gene function. A knock-out of the *Sgpl1* gene was employed to investigate the efficacy of an Adeno-associated virus (AAV) therapeutic strategy for S1P lyase insufficiency syndrome (82). AAV9-mediated transfer of human Sphingosine-1-phosphate lyase 1 (SGPL1) delivered to newborn *Sgpl1* knockout (KO) mice, which normally die in the first few weeks of life, led to prolonged survival and ameliorated pathology, including neurodevelopmental delay, anaemia and hypercholesterolaemia. However, for dominant disorders, approaches which involve allele-specific activation are required such as RNA interference (RNAi) allele-specific knockdown or editing approaches. A novel mouse model carrying a targeted dominant, small deletion allele of Charcot–Marie–Tooth disease type 2D demonstrated typical phenotypic features, including early onset neuropathy, that validated the human allele and was used to assess allele-specific RNAi for effective gene therapy (83). RNAi sequences targeting the mutant allele, but not wild-type, delivered by AAV9 at birth prevented development of the neuropathy. CRISPR/Cas9 has also been successfully employed using allele-specific approaches to disrupt the dominant mutant allele, *Beethoven*, in the *Tmc1* gene that leads to progressive hearing loss. Two delivery systems were employed, either injection of Cas9:guide RNA:lipid complexes into the cochlea of mutant mice (84), or the use of an AAV vector delivering a protospacer-adjacent motif (PAM) variant of *Staphylococcus aureus* Cas9 (SaCas9-KKH) that selectively and efficiently disrupted the mutant allele (85). Base editing also shows potential for gene therapy, and mouse models will critically assist in their assessment. Hutchinson–Gilford progeria syndrome caused by a dominant-negative C-G to T-A mutation (c.1824 C > T; p.G608G) in Lamin A (LMNA) has been the focus of a recent effort. The delivery via retro-orbital injection of AAV9 encoding adenine base editors to mouse transgenics homozygous for the human mutant gene was effective at ameliorating vascular pathology and substantially extending lifespan (86).

## Conclusion

Over the last 30 years, the mouse genetic toolbox from mutant generation to pathophysiology has had a transformative impact on the biomedical sciences, from basic mechanistic studies elucidating genetic phenomena to the development of therapeutic approaches. Critically, the mouse maintains a unique position in the dissection of mammalian *in vivo* mechanisms and systems for the elaboration of pathophysiology and disease, which are not amenable through *in vitro* cellular and organoid studies, or human genetics approaches. The mouse toolbox is in continual flux with new approaches, techniques and resources constantly emerging. Mouse genetics is accelerating our pan-genomic understanding of the genetic landscape of disease and is providing an extraordinary knowledge base for the many challenges we face in tackling the vision of genomic and precision medicine.
